# Pleiotropic Effects of PCSK9 Inhibitors on Cardio-Cerebrovascular Diseases

**DOI:** 10.3390/biomedicines12122729

**Published:** 2024-11-28

**Authors:** Zhenzhen Li, Lin Zhu, Yeqiong Xu, Yiting Zhang, Yukai Liu, Huiling Sun, Shuo Li, Meng Wang, Teng Jiang, Junshan Zhou, Qiwen Deng

**Affiliations:** 1 Department of Neurology, Nanjing First Hospital, Nanjing Medical University, Nanjing 210006, China; zhenzhenli0304@163.com (Z.L.); julinzz@163.com (L.Z.); zhangyt@stu.njmu.edu.cn (Y.Z.); lyk8787@126.com (Y.L.); lishuosunshine@sina.com (S.L.); 15895826278@163.com (M.W.); jiang_teng@njmu.edu.cn (T.J.); 2 Central Laboratory of Changshu Medical Examination Institute, Changshu 215500, China; xuyeqiong.hi@163.com; 3 General Clinical Research Center, Nanjing First Hospital, Nanjing Medical University, Nanjing 210006, China; sunhuiling1988@yeah.net

**Keywords:** pleiotropic effects, proprotein convertase subtilisin-kexin type 9, central nervous system, cardiovascular system, lipid metabolism, atherosclerosis, inflammation, platelet

## Abstract

Cardiovascular disease (CVD) and ischemic stroke (IS) are the primary causes of mortality worldwide. Hypercholesterolemia has been recognized as an independent risk factor for CVD and IS. Numerous clinical trials have unequivocally demonstrated that reducing levels of low-density lipoprotein cholesterol (LDL-C) significantly mitigates the risk of both cardiac and cerebral vascular events, thereby enhancing patient prognosis. Consequently, LDL-C reduction remains a pivotal therapeutic strategy for CVD and IS. However, despite intensive statin therapy, a significant proportion of high-risk hypercholesterolemic patients fail to achieve sufficient reductions in LDL-C levels. In response to this challenge, an inhibitor targeting proprotein convertase subtilisin-kexin type 9 (PCSK9) has been developed as a therapeutic intervention for hyperlipidemia. Numerous randomized controlled trials (RCTs) have conclusively demonstrated that the combination of PCSK9 inhibitors and statins significantly enhances prognosis not only in patients with CVD, but also in those afflicted with symptomatic intracranial artery stenosis (sICAS). PCSK9 inhibitors significantly reduce LDL-C levels by binding to the PCSK9 molecule and preventing its interaction with LDLRs. This prevents degradation of the receptor and increases uptake of LDL-C, thereby decreasing its concentration in blood. Besides significantly reducing LDL-C levels, PCSK9 inhibitors also demonstrate anti-inflammatory and anti-atherosclerotic properties while promoting plaque stabilization and inhibiting platelet aggregation and thrombosis. This article aims to provide a comprehensive review based on the relevant literature regarding the evolving understanding of pleiotropic effects associated with PCSK9 inhibitors, particularly focusing on their impact on the cardiovascular system and central nervous system.

## 1. Introduction

Cardiovascular disease (CVD) and ischemic stroke (IS) are the primary causes of mortality worldwide [1,2]. Hypercholesterolemia has been recognized as an independent risk factor for CVD and IS [3,4,5]. Numerous clinical trials have unequivocally demonstrated that reducing levels of low-density lipoprotein cholesterol (LDL-C) significantly mitigates the risk of both cardiac and cerebral vascular events, thereby enhancing patient prognosis. Consequently, LDL-C reduction remains a pivotal therapeutic strategy for CVD and IS. For decades, statins have been regarded as the primary therapeutic option for reducing cholesterol levels and preventing potential cardiovascular and cerebrovascular events. However, despite intensive statin therapy, a significant proportion of high-risk hypercholesterolemic patients fail to achieve sufficient reductions in LDL-C levels [6,7]. In response to this challenge, an inhibitor targeting proprotein convertase subtilisin-kexin type 9 (PCSK9) has been developed as a therapeutic intervention for hyperlipidemia, effectively achieving a significant reduction in low-density lipoprotein cholesterol (LDL-C) levels within a month. Numerous randomized controlled trials (RCTs) have conclusively demonstrated that the combination of PCSK9 inhibitors and statins significantly enhances prognosis not only in patients with CVD but also in those afflicted with symptomatic intracranial artery stenosis (sICAS). In this study, we aim to provide a comprehensive overview of the pleiotropic effects of PCSK9 inhibitors.

**Figure 1:**
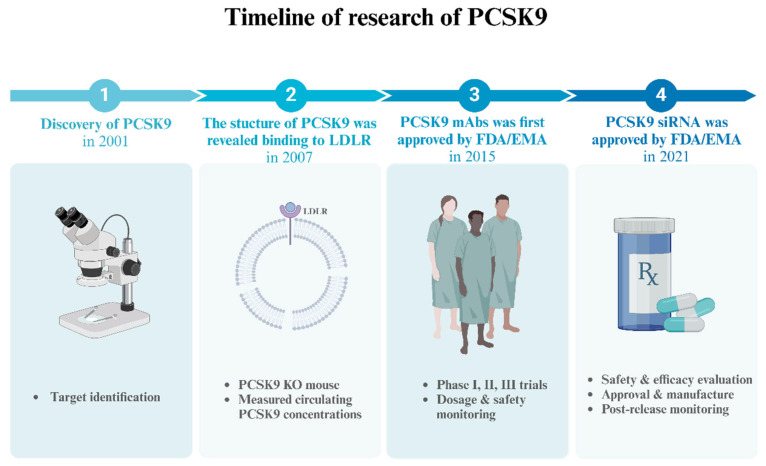
**Timeline of research on PCSK9.** In 2001, PCSK9 was initially discovered. In 2007, the structure of PCSK9 was revealed binding to LDLR. In 2015, PCSK9 mAbs was first approved by FDA/EMA. Subsequently, a series of phase I, II, and III trials were conducted. In 2021, PCSK9 siRNA was approved by FDA/EMA. Abbreviations: PCSK9, proprotein convertase subtilisin-kexin type 9. LDLR, low-density lipoprotein cholesterol receptor. PCSK9 KO, PCSK9 knockout. PCSK9 mAbs, monoclonal antibodies against PCSK9. FDA, the Food and Drug Administration. EMA, the European Medicines Agency. PCSK9 siRNA, PCSK9 small interfering RNA. The figure was created with BioRender.com.

**Figure 2:**
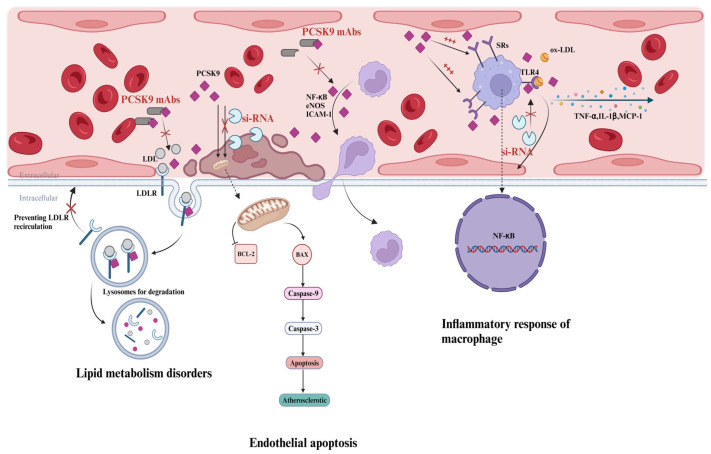
**PCSK9 inhibitors in lipid metabolism and anti-atherosclerotic and anti-inflammatory effects.** First, PCSK9 binds to the LDLR, leading to a reduction in LDLR expression. Consequently, the ability to eliminate LDL-C diminishes. PCSK9 mAbs significantly reduce LDL-C levels by binding to the PCSK9 molecule and preventing its interaction with the LDLR. This prevents degradation of the receptor and increases uptake of LDL-C, thereby decreasing its concentration in blood. Second, PCSK9 siRNA effectively inhibits endothelial cell apoptosis by reducing the expression of pro-apoptotic proteins such as Bax, Caspase3, and Caspase9. Conversely, it is observed that the level of anti-apoptotic protein Bcl-2 is increased, ultimately reducing the development of atherosclerosis. Third, PCSK9 mAbs effectively suppress monocyte adhesion to endothelial cells through reducing the expression of NF-κB, eNOS, and ICAM-1. Ultimately, this leads to a decrease in both the size of atherosclerotic plaques formed as well as an attenuation of the inflammatory response. In addition, macrophage inflammatory responses induced by ox-LDL play a pivotal role in the pathogenesis of atherosclerosis. PCSK9 may accelerate inflammation of atherosclerotic plaques through activating the TLR 4/NF-κB pathway and promoting interactions between SRs on macrophage surfaces. PCSK9 siRNA reduces macrophage numbers and attenuates inflammatory response triggered by ox-LDLs in macrophages. Consequently, there is a subsequent decrease in the expression of vascular inflammation regulators such as TNF-α, IL-1β, and MCP-1. Abbreviations: PCSK9, proprotein convertase subtilisin-kexin type 9. LDL, low-density lipoprotein cholesterol. LDLR, low-density lipoprotein cholesterol receptor. PCSK9 mAbs, monoclonal antibodies against PCSK9. siRNA, small interfering RNA. NF-κB, nuclear factor kappa-B. eNOS, endothelial nitric oxide synthase. ICAM-1, intercellular cell adhesion molecule-1. ox-LDL, oxidized low-density lipoprotein. TLR 4, Toll-like receptor 4. TNF-α, tumor necrosis factor-alpha. IL-1β, interleukin 1 beta. MCP-1, monocyte chemoattractant protein-1. The figure was created with BioRender.com.

In 2001, PCSK9 was initially discovered when elevated protein levels were found in studies on cerebellar neuron apoptosis (Figure 1). Subsequently, in 2003, the protein was named neural apoptosis-regulated convertase 1. The gene responsible for PCSK9 is located on chromosome 1p32 and encodes a serine protease comprising 692 amino acids [8]. PCSK9 is primarily expressed in the liver, with lower levels of protein expression also observed in the intestine, kidney, and select regions of the central nervous system [9]. As it circulates in the bloodstream, PCSK9 binds to the low-density lipoprotein cholesterol receptor (LDLR) present on hepatocyte surfaces, leading to a reduction in LDLR expression. Consequently, the hepatocytes’ ability to eliminate LDL-C diminishes. It has been found that PCSK9 inhibitors significantly reduce LDL-C levels by binding to the PCSK9 molecule and preventing its interaction with the LDLR. This prevents degradation of the receptor and increases uptake of LDL-C, thereby decreasing its concentration in blood [10] (Figure 2).

Currently, the primary types of PCSK9-targeted inhibitors include monoclonal antibodies against PCSK9 (PCSK9 mAbs), small interfering RNAs, antisense oligonucleotides (ASOs) and other emerging PCSK9 inhibitors, such as vaccines against PCSK9, oral PCSK9 inhibitors, and gene therapy (Table 1). In clinical practice, monoclonal antibodies are widely utilized, specifically alirocumab, evolocumab, and bococizumab. These PCSK9 mAbs competitively bind to both PCSK9 and LDLR, effectively blocking their interaction. Inclisiran is a small interfering RNA (siRNA) that suppresses the expression of the *PCSK9* gene, leading to an increased number of LDLRs and enhanced clearance of LDL-C from the bloodstream. Consequently, this mechanism ultimately lowers LDL-C levels. Recent research has revealed diverse effects of PCSK9 inhibitors across various systems and organs. This article aims to provide a comprehensive review based on the relevant literature regarding the evolving understanding of pleiotropic effects associated with PCSK9 inhibitors, particularly focusing on their impact on the cardiovascular system and central nervous system.

## 2. Enhanced Application of PCSK9 Inhibitors in the Cardiovascular and Cerebrovascular Systems

### 2.1. Involvement of PCSK9 Inhibitors in Lipid Metabolism

LDL-C has been identified as the primary risk factor for atherosclerosis, which is a significant contributing factor to the development of atherosclerotic cardiovascular and cerebrovascular disease [11,12]. The prevalence of atherosclerotic cardiovascular disease (ASCVD) and IS in China has been consistently increasing on an annual basis, presenting ongoing challenges in the management of lipid disorders.

PCSK9 plays a crucial role in maintaining cholesterol and lipid homeostasis. It directly binds to the epidermal growth factor precursor homology domain A (EGF-A) of LDLR, guiding the receptor towards intracellular lysosomes for degradation and preventing its recirculation to the cell surface [19,20]. In recent years, numerous clinical studies have demonstrated the effectiveness of PCSK9 inhibitors in significantly reducing cardiovascular and cerebrovascular disease by markedly lowering LDL-C levels. The FOURIER trial [21] with a median follow-up of 2.2 years and the ODYSSEY OUTCOMES study [22] showed that PCSK9 inhibitors (evolocumab or alirocumab) effectively lowered LDL-C levels and significantly reduced major cardiovascular events over a median follow-up of 2.2 years. Similar results were seen in the latest FOURIER-OLE study, which had a maximum follow-up duration of 8.6 years [23]. In terms of safety-specific or overall adverse events, no significant differences were observed [21,22]. However, there was a 15% reduction in major adverse cardiovascular events (MACEs) and all-cause mortality following acute coronary syndrome [22]. The clinical trial evaluating the efficacy of inclisiran in ASCVD patients over a period of 5 years demonstrated a significant and sustained reduction of 44.2% in LDL-C levels within the inclisiran group. Mild injection site events were the only treatment-related adverse reactions observed [24]. Moreover, patients demonstrated improved adherence with biannual administration of inclisiran compared to subcutaneous administration of PCSK9 monoclonal antibodies every two weeks. Additionally, findings from the FOURIER and ODYSSEY OUTCOMES clinical trials demonstrated a significant reduction in the risk of cerebrovascular disease, encompassing both ischemic stroke and recurrent ischemic stroke, with the utilization of PCSK9 inhibitors.

However, regarding the adverse events, the incidence of hemorrhagic stroke was noted to be less than 0.1% in the alirocumab group and 0.2% in the placebo group in the ODYSSEY OUTCOMES trial, which shows no statistically significant difference [11]. Similarly, the same results were observed in the FOURIER trial [12]. A meta-analysis comprising 16 RCTs revealed a noteworthy 23% reduction in the risk of ischemic stroke after using PCSK9 inhibitors [25]. This finding was further supported by an additional meta-analysis that included an extensive pool of 35 RCTs [26]. The findings of a multicenter prospective cohort study demonstrate that patients with ICAS who received PCSK9 inhibitors in addition to statin therapy showed lowered LDL-C levels and exhibited a reduced incidence rate of early recurrent stroke [27], highlighting the potential benefits of this combination treatment.

PCSK9 also influences the metabolism of Lp(a), an independent risk factor for cardiovascular and cerebrovascular events [28]. Lp(a) is a type of LDL particle consisting of apolipoprotein(a) (apo(a)) covalently linked to apolipoprotein B-100 (apoB) [29]. On the one hand, PCSK9 inhibitors increased clearance of Lp(a) particles via related receptors (such as LDLRs, LRP receptors, plasminogen receptors, CD36, TLR2, and scavenger receptor-B1). On the other hand, PCSK9 inhibitors decreased Lp(a) production [30]. A combined analysis of four phase II trials evaluating evolocumab revealed a dose-dependent reduction in Lp(a) levels ranging from 25% to 29% [31]. Patients with higher baseline Lp(a) levels demonstrated more significant absolute reductions in Lp(a) and a greater propensity to derive coronary benefits from PCSK9 inhibition. Similar findings were observed in the FOURIER trial. Furthermore, a study conducted among normolipidemic individuals reported that evolocumab also decreased plasma Lp(a) concentration by reducing the production and accelerating the catabolism of Lp(a) particles [32]. This enhancement in catabolism is likely attributed to the significant upregulation of hepatic receptors, primarily those for LDL, and/or a reduction in the competition between Lp(a) and LDL particles for these receptors [33]. However, the elaborate mechanisms of the involvement of PCSK9 and its inhibitors in Lp(a) metabolism still need to be explored by further studies.

Very-low-density lipoprotein (VLDL) is produced in the liver as the endogenous lipoprotein and metabolized to LDL after secretion. PCSK9 can contribute to the degradation of the very-low-density lipoprotein cholesterol receptor (VLDLR) independently of LDLR [34,35,36]. Compared with wild-type (WT) mice, PCSK9 knockout mice exhibited a remarkable 40-fold elevation in VLDLR levels. Additionally, there was a 36-fold increase in VLDLR levels observed in LDLR and PCSK9 knockout mice [34]. Similarly, Poirier et al. found that both VLDLR and ApoER2 can directly or indirectly interact with PCSK9 independent of LDLR [19]. In a real-world study, the size of VLDL increases after treatment with PCSK9 inhibitors, while that of VLDL-associated lipoproteins decreases [37]. Moreover, inhibition of PCSK9 with evolocumab markedly reduced VLDL particle concentrations in addition to lowering LDL-C. The extent of reduction in VLDL particles depended on the baseline level of Lp(a) [38]. Regarding the potential mechanism, studies related to the structure of VLDLR have identified an EGF-A domain which is homologous to that found in LDLR. As a result, the mechanism of PCSK9 in regulating VLDLR appears to be similar to that observed in LDLR regulation. Specifically, PCSK9 interacts with VLDLR by binding to the EGF-A domain, forming an endocytic complex which is degraded through the lysosomal pathway and ultimately regulating the levels of VLDLR [35]. But the precise mechanism of interaction between PCSK9 and VLDLR remains unclear. Moreover, PCSK9 can induce degradation of low-density lipoprotein receptor-related protein 1 (LRP-1). Although PCSK9 exhibits the capability to trigger LRP-1 degradation, LRP-1 is not an essential component for LDLR regulation. Furthermore, LDLR actively competes with LRP-1 for interaction with PCSK9 [39]. However, the specific relationship between PCSK9 and the LRP receptor family remains incompletely understood.

### 2.2. Involvement of PCSK9 Inhibitors in Anti-Atherosclerotic and Plaque-Stabilizing Effects

Atherosclerosis is a prevalent chronic cardiovascular disease characterized by disturbances in lipoprotein metabolism and vascular inflammation, with LDL-C playing a crucial role as a component of plasma lipoproteins. The utilization of PCSK9 inhibitors has demonstrated significant reductions in cardiovascular and cerebrovascular events through the lowering of LDL-C levels. Moreover, beyond this primary mechanism, PCSK9 inhibitors may exert additional effects on atherogenesis through alternative pathways.

Endothelial injury is a crucial initiator of atherosclerosis. Extensive apoptosis of endothelial cells can disrupt the integrity and increase the permeability of the endothelium, thereby promoting endothelial dysfunction and atherogenesis. In apoptotic human umbilical vein endothelial cells(HUVECs), both the mRNA and protein expression levels of PCSK9 were significantly elevated compared to normal cells [40]. Cellular experiments demonstrated that PCSK9 siRNA effectively inhibited HUVEC apoptosis by reducing the expression of pro-apoptotic proteins such as Bax, Caspase3, and Caspase9. Conversely, it was observed that the level of anti-apoptotic protein Bcl-2 was increased [40]. Among these proteins, Bax acts as a pro-apoptotic factor by enhancing outer mitochondrial membrane permeability through pore formation. On the other hand, Bcl-2 plays an opposite role as an anti-apoptotic protein with its ratio to Bax indicating an apoptotic susceptibility ability. It suggested that PCSK9 modulates apoptosis-associated proteins to facilitate endothelial cell apoptosis, ultimately contributing to the development of atherosclerosis. Moreover, short hairpin RNA targeting PCSK9 effectively suppressed both the mRNA and protein levels of PCSK9, while significantly attenuating the phosphorylation of p38 and c-Jun N-terminal kinases [41]. Additionally, in human coronary artery endothelial cells, evolocumab and alirocumab effectively suppress monocyte adhesion to endothelial cells through the NF-κB and eNOS signaling pathways [42] (Figure 2).

The PCSK9 inhibitors not only exhibit a significant reduction in LDL-C levels but also demonstrate the potential for plaque regression. Analysis of intravascular ultrasound images of abdominal arteries from rabbits fed an atherosclerotic diet at weeks ten and eighteen revealed that both alirocumab and atorvastatin effectively reduced the percentage of atherosclerotic plaque volume. However, in terms of inhibiting the progression and stability of atherosclerotic plaques in rabbits, evolocumab exhibited higher absolute values for regression coefficients in percent atheroma volume, fibrotic composition, and necrotic composition compared to atorvastatin [43]. Furthermore, in the GLAGOV clinical trial, a significantly higher proportion of patients receiving evolocumab exhibited plaque regression compared to the control group, accompanied by a noteworthy reduction in both the percentage of atherosclerotic plaque volume and standardized total atherosclerotic volume [44]. Additionally, in the early stages of acute ACS, the addition of evolocumab to statin therapy may result in incremental enhancement of fibrous cap thickness and regression of lipid-rich plaque, potentially resulting in a more pronounced reduction in LDL-C levels [45]. High-resolution MRI (HRMRI) was conducted to assess the impact of PCSK9 inhibitors on intracranial plaques in ICAS patients receiving moderate-intensity statin therapy. Following a 12-week follow-up, the PCSK9 inhibitor group exhibited significant reductions in both the standardized wall index and degree of stenosis compared to the statin group [46]. Furthermore, patients treated with alirocumab demonstrated a gradual decrease in plaque lipid content, accompanied by an increase in fibrous tissue proportion and degradation of the neovascularization system as measured by MRI, indicating that PCSK9 inhibitors also contribute to carotid plaque stabilization [47]. Therefore, the inhibition of endothelial cell apoptosis by PCSK9 inhibitors may potentially mitigate atherosclerosis and induce plaque regression.

### 2.3. Involvement of PCSK9 Inhibitors in Anti-Inflammatory Effects

The pathogenesis of atherosclerosis involves chronic inflammation, characterized by infiltration of macrophages and progressive accumulation of inflammatory mediators and oxidative stress in the arterial wall, ultimately leading to the development of cardiovascular diseases associated with atherosclerosis.

Macrophage inflammatory responses induced by oxidized low-density lipoprotein (ox-LDL) play a pivotal role in the pathogenesis of atherosclerosis. In an apoE knockout mouse model, compared to the control group, mice treated with lentivirus-mediated PCSK9 shRNA exhibited reduced macrophage numbers and attenuated inflammatory response triggered by ox-LDLs in macrophages. Consequently, there was a subsequent decrease in the expression of vascular inflammation regulators such as tumor necrosis factor-alpha (TNF-α), interleukin 1 beta (IL-1β), monocyte chemoattractant protein-1 (MCP-1), Toll-like receptor 4 (TLR 4), and nuclear factor-kappa B (NF-κB). It is hypothesized that activation of the TLR 4/NF-κB pathway may serve as a potential mechanism for accelerated inflammation of atherosclerotic plaques influenced by PCSK9 [48]. Additionally, PCSK9 promotes interactions between scavenger receptors (SRs), including LOX-1 and CD36, on macrophage surfaces. This concurrent stimulation leads to downstream activation of inflammation-related markers and subsequent exertion of anti-inflammatory effects [49,50]. In a mouse model of hyperlipidemia with high PCSK9 expression, there was an observed increase in monocyte infiltration within the vessel wall. In a murine model exhibiting hyperlipidemia and elevated levels of PCSK9 expression, an observable augmentation in monocyte infiltration within the vascular wall was noted [51]. Additionally, treatment with the AT04A anti-PCSK9 vaccine resulted in a significant reduction in plasma inflammatory markers including serum amyloid A, macrophage inflammatory protein-1β, macrophage-derived chemokine, cytokine stem cell factor, and vascular endothelial growth factor A (VEGF-A). Macrophage colony-stimulating factor 1 (M-CSF-1) and VEGF-A induce activation of endothelial cells and promote migration of monocytes/macrophages. As a result, decreased levels of M-CSF-1 and VEGF-A lead to reduced expression of ICAM-1 in endothelial cells, thereby reducing monocyte recruitment and adhesion to the vascular endothelium. Ultimately, this leads to a decrease in both the size of the atherosclerotic plaques formed as well as an attenuation of the inflammatory response [52] (Figure 2). Similarly, another study conducted on mice demonstrated that treatment with alirocumab led to decreased levels of ICAM-1 expression as well as other markers associated with vascular inflammation [53]. Furthermore, PCSK9 inhibitors may not only decrease pro-inflammatory mediators but also enhance vascular inflammation by upregulating anti-inflammatory cytokines such as IL-10 [54,55].

Furthermore, a subgroup analysis conducted in the FOURIER clinical study has highlighted the particularly noteworthy clinical benefit of using PCSK9 mAbs in patients with heightened baseline inflammatory markers, such as high-sensitivity C-reactive protein (hs-CRP) [56]. It explored the efficacy of evolocumab stratified by baseline high-sensitivity C-reactive protein (hs-CRP) (<1, 1–3, and >3 mg/dL). And the result showed that, in the evolocumab arm, the absolute risk reductions for the primary and key secondary end points tended to be greater in patients with higher hs-CRP. Patients in the alirocumab-treated group demonstrated significantly reduced lipid levels and diminished arterial wall inflammation as assessed by 18F-fluorodeoxyglucose positron emission tomography/computed tomography compared to placebo controls, while circulating inflammatory markers remained unaffected [57]. Additionally, patients treated with PCSK9 inhibitors exhibited reduced expression of pro-inflammatory proteins and increased levels of SIRT3 and collagen within the plaque [58]. These results were obtained despite comparable circulating hs-CRP levels and were also observed in LDL-C-matched subgroups with LDL-C levels < 100 mg/dL. It suggests that the use of PCSK9 inhibitors is associated with a favorable remodeling of the inflammatory burden within the human atheroma, potentially independent or partially independent of their ability to lower LDL-C. However, due to factors such as shorter follow-up periods and limited participant enrollment, there remains a scarcity of high-quality evidence supporting these findings regarding the anti-inflammatory role of PCSK9 mAbs in clinical trials.

### 2.4. Involvement of PCSK9 Inhibitors in Antiplatelet Aggregation and Antithrombosis

Platelets are widely recognized for their pivotal role in the initial stages of atherosclerotic plaque formation. Following the rupture of an unstable atherosclerotic plaque, substantial quantities of procoagulant factors are released into the vessel lumen, thereby instigating the development of atherosclerotic thrombosis and subsequently triggering cardiovascular events. Notably, platelets and fibrinogen, both crucial components in thrombus formation, exhibit a robust positive correlation with PCSK9 [59,60].

**Figure 3:**
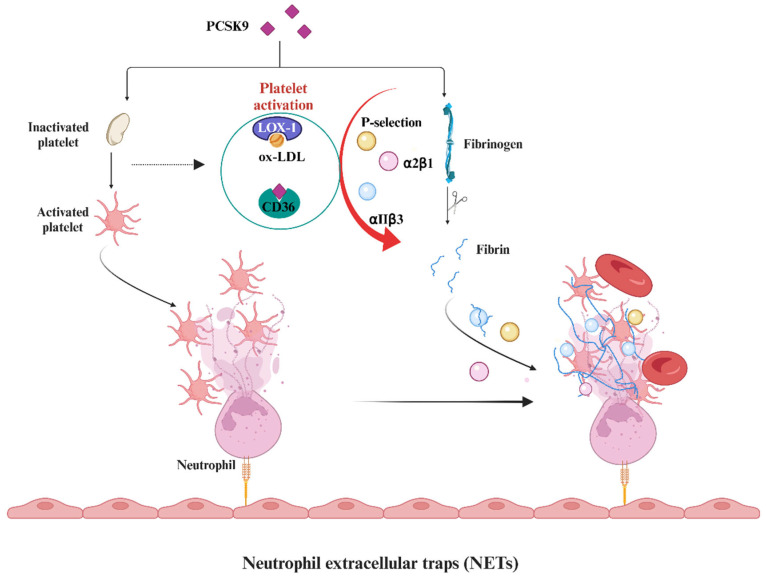
**PCSK9 inhibitors in antiplatelet aggregation and antithrombosis**. PCSK9 activates CD36 and LOX-1 receptors in platelets, thereby enhancing platelet activation. The interaction between CD36 and ox-LDL triggers signaling pathways, leading to the expression of P-selectin and activation of integrin αIIb β3 (the receptor for fibrinogen). This promotes the formation of platelet–leukocyte complexes through P-selectin and cross-linking adjacent platelets via fibrinogen. Additionally, the binding of ox-LDL to LOX-1 triggers the activation of integrins αIIbβ3 and α2β1, subsequently inducing alterations in platelet morphology and aggregation, ultimately facilitating thrombosis. In addition, deficiency in PCSK9 is associated with protection against venous thrombosis by reducing leukocyte recruitment and NET formation at the site of thrombosis. Abbreviations: PCSK9, proprotein convertase subtilisin-kexin type 9. ox-LDL, oxidized low-density lipoprotein. CD36, platelet glycoprotein 4, a scavenger receptor (SR). LOX-1, lectin-like oxidized low-density lipoprotein receptor-1. αIIbβ3 and α2β1, the receptors for fibrinogen. The figure was created with BioRender.com.

The plasma level of PCSK9 was found to be positively correlated with platelet counts in patients with coronary artery disease, suggesting that the involvement of PCSK9 in atherosclerosis includes platelet-related mechanisms [61]. Subsequently, it was observed in in vivo experiments that PCSK9 activates CD36 and LOX-1 receptors in platelets, thereby enhancing platelet activation [59,62]. The interaction between CD36 and ox-LDL triggers signaling pathways that activate platelets, leading to the expression of P-selectin and activation of integrin αIIb β3 (the receptor for fibrinogen). This promotes the formation of platelet–leukocyte complexes through P-selectin and cross-linking adjacent platelets via fibrinogen. Additionally, the binding of ox-LDL to LOX-1 triggers the activation of integrins αIIbβ3 and α2β1, subsequently inducing alterations in platelet morphology and aggregation, ultimately facilitating thrombosis [63]. Furthermore, compared to WT mice, PCSK9-deficient mice exhibited a reduced incidence of venous thrombosis following ligation of the inferior vena cava, as well as decreased neutrophil extracellular trap (NET) formation and leukocyte attachment [64]. In summary, deficiency in PCSK9 is associated with protection against venous thrombosis by reducing leukocyte recruitment and NET formation at the site of thrombosis (Figure 3).

The effect of PCSK9 inhibition on venous thromboembolism (VTE) was investigated through a meta-analysis that included the FOURIER [12] and ODYSSEY OUTCOMES [11] studies. The analysis revealed a significant 31% reduction in VTE risk with PCSK9 inhibition, while no correlation was found between baseline LDL-C levels and VTE risk reduction. However, a noteworthy interaction was observed between baseline Lp(a) concentration and VTE risk reduction, suggesting the potential of Lp(a) inhibitors [65]. Furthermore, a prospective study encompassing 24 patients with hypercholesterolemia with PCSK9 mAbs over a 12-month follow-up revealed a decrease in platelet aggregation and activation biomarkers, including soluble CD40 ligand, platelet factor-4, and soluble P-selectin. These findings suggest that PCSK9 inhibitors could potentially contribute to a reduction in cardiovascular events [66]. Nevertheless, further research is warranted to elucidate the effects and mechanisms of PCSK9 inhibitors on platelet function and their antithrombotic potential.

## 3. PCSK9 Inhibitors and the Central Nervous System

In recent years, the involvement of PCSK9 in the central nervous system has garnered significant attention, in addition to its established role in cardiovascular systems. Initially discovered to play a pivotal role in neurodevelopment, PCSK9 knockout experiments on zebrafish demonstrated a profound disruption of cerebellar neurons and loss of hindbrain–midbrain boundaries, ultimately leading to embryonic demise at 96h post-fertilization [67]. Interestingly, silencing PCSK9 is non-lethal in mammals such as mice or humans, as evidenced by the survival of PCSK9 knockdown mice with intact telencephalic and cerebellar tissues [68]. In relation to PCSK9 inhibitors and ischemic stroke, animal experiments demonstrated an upregulation of PCSK9 transcripts in the dentate gyrus of the hippocampus on the injured side following transient middle cerebral artery occlusion, suggesting a potential role of endogenous PCSK9 in cerebral ischemic injury [68]. Separate investigations revealed that PCSK9 inhibitors effectively suppressed the activation of cysteinyl asparagin-3 and c-Jun potassium-deprived cerebellar granule neurons. Further mechanistic exploration unveiled ApoER2 as a potential pivotal target for mitigating neuronal apoptosis during this specific process using PCSK9 inhibitors [36]. Moreover, administration of alirocumab to mice fed a high-fat–cholesterol diet resulted in significantly reduced expression levels of LRP-1, BACE1, and Aβ42 in the hippocampus. Moreover, cognitive deficits were ameliorated. Additionally, alirocumab-treated mice exhibited decreased levels of various inflammatory mediators including NF-κB, TNF-α, IL-1β, IL-6, and TLR4, indicating potential anti-inflammatory effects of the drug [69].

The use of PCSK9 inhibitors, similar to statins [70] in cognitive function, did not demonstrate an increased risk of cognitive impairment [11]. A meta-analysis indicated an apparently higher incidence of these adverse events in the ODYSSEY and OSLER trials, but found no significant difference overall [71]. Additionally, a study evaluating 2,086 patients for Spatial Working Memory Strategies (SWMSs) found that a change from baseline to week 96 in SWMS z-score achieved noninferiority between alirocumab and placebo (least squares mean change at week 96, −0.180 vs. −0.200). Exploratory outcome measures, which further assessed neurocognitive function in the Cambridge Neuropsychological Test Automated Battery (CANTAB) domains, did not differ significantly over 96 weeks [72]. Furthermore, the MEMOGAL study with a follow-up period of up to 24 months demonstrated no significant effect on neurocognitive function by PCSK9 inhibitors in terms of global MoCA score or different cognitive domains. However, for delayed recall memory, the mean change was statistically significant (improvement) + 0.44 [73]. To investigate the potential influence of the APOE genotype on the association between evolocumab usage and cognitive function, Korthauer et al. enrolled 13,481 patients with available APOE genotyping data from the FOURIER trial, among whom 28% were carriers of the ε4 allele. After a two-year treatment and monitoring period, they found no significant impact of the APOE genotype on the relationship between the treatment group and performance on the CANTAB. This discovery provides valuable insights for future research exploring the potential benefits of cholesterol-lowering medications in individuals genetically predisposed to Alzheimer’s disease [74].

In conclusion, aside from significantly reducing cholesterol levels, PCSK9 inhibitors may confer beneficial effects on the recurrence of ischemic stroke and cognitive impairment. However, a comprehensive exploration is still needed to elucidate the precise effects and mechanisms by which PCSK9 inhibitors act in the central nervous system (CNS). Therefore, further clinical data are essential to establish a more robust foundation for clarifying the role and safety profile of PCSK9 inhibitors in treating CNS diseases.

## 4. PCSK9 Inhibitors and Other Systems

CVD is one of the most common complications and stands as the predominant cause of mortality among individuals diagnosed with diabetes mellitus (DM) [75]. Dyslipidemia is one of the important risk factors for CVD. Therefore, effective management of blood lipids is a crucial measure for controlling cardiovascular risk factors in DM patients. The use of statins to lower LDL-C represents a cornerstone in the management of cardiovascular risk among individuals with type 2 DM (T2DM) [75]. However, some studies indicate that statin therapy increases the rate of diabetes development in individuals without diabetes or shows a moderate but significant impact on glycemia progression in patients with T2DM and people at high risk of diabetes (including obesity, overweight, and metabolic disorders) [76,77]. Therefore, it is not clear whether there similar exist with the use of PCSK9 inhibitors or not. In mouse β−cells, PCSK9 deficiency does not influence insulin secretion or the islet content of cholesterol, indicating that PCSK9 inhibition may not exacerbate diabetes [78,79]. However, Mendelian randomization studies indicate that PCSK9 loss-of-function variants can reduce serum LDL-C while increasing fasting glucose levels and the risk of T2DM [80]. In summary, studies in pathophysiology and genetics indicate that PCSK9 inhibition may modestly elevate the risk of new-onset diabetes. However, results from large clinical trials suggest that PCSK9 inhibitor treatment does not increase the risk of diabetes [22]. Furthermore, studies have found that PCSK9 mutations are associated with elevated fasting glucose levels, increased body weight, an elevated waist-to-hip ratio, and an increased risk of T2DM [80]. However, despite the existing research, there remains a scarcity of large-scale clinical trials conclusively demonstrating the link between PCSK9 inhibition and metabolic disorders such as obesity, overweight, and hypertension. In addition, there have been relevant studies on PCSK9 inhibition in sepsis [81], viral infections [82], and cancer [83], but the specific mechanisms are still unclear.

**Figure 4:**
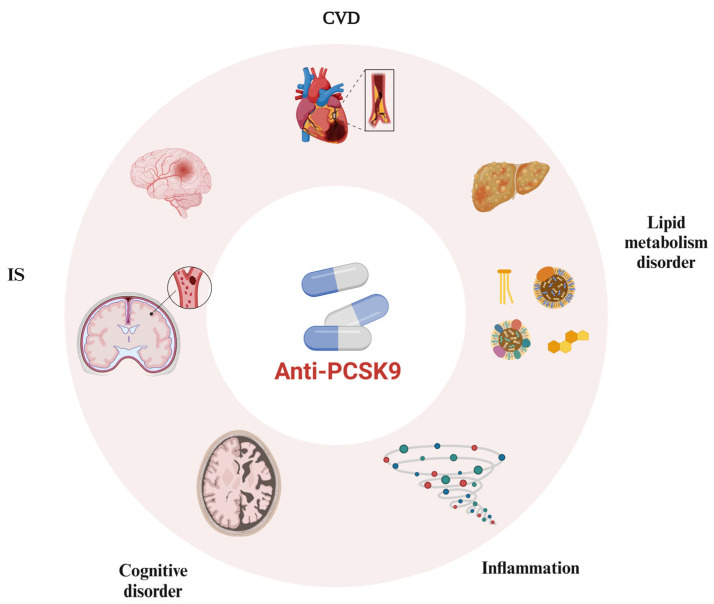
**Pleiotropic effects of PCSK9 inhibitors on cardio-cerebrovascular diseases**. PCSK9 inhibitors not only influence lipid metabolism but also exert effects on the cardiovascular system, central nervous system, and other physiological processes. Abbreviations: PCSK9, proprotein convertase subtilisin-kexin type 9. CVD, cardiovascular disease. IS, ischemic stroke. The figure was created with BioRender.com.

Overall, as research progresses, PCSK9 not only disrupts lipid metabolism but also exerts effects on the cardiovascular system, central nervous system, and other physiological processes. Similarly, besides significantly reducing LDL-C levels, PCSK9 inhibitors also demonstrate anti-inflammatory and anti-atherosclerotic properties while promoting plaque stabilization and inhibiting platelet aggregation and thrombosis (Figure 4). However, further studies are required to explore the cognitive effects and underlying mechanisms of evolocumab and alirocumab. Complex associations and interactions may exist between PCSK9 expression in various organs and systems, necessitating further investigation into the specific mechanisms and linkages. Additionally, the safety considerations surrounding PCSK9 inhibitors warrant more prospective clinical studies and a substantial amount of clinical data to establish their efficacy. It is anticipated that with an increasing number of high-quality clinical studies and deeper exploration of molecular mechanisms through basic research, the application of PCSK9 inhibitors will continue to expand, benefiting a larger population of patients.

## Figures and Tables

**Table 1:** Therapies targeting PCSK9 in recent development.

Types	Drug Name	Description	Trials	Clinical Trial Name	Clinical Trial Phase	Mechanism
**PCSK9 mAbs**	Alirocumab [11]	Monoclonal antibodies	ODYSSEY OUTCOMES	NCT01663402	3	Targeting to bind with PCSK9
	Evolocumab [12]		FOURIER	NCT01764633	3	
	Bococizumab [13]		SPIRE	NCT01975376 NCT01975389	3 *	
**siRNA**	Inclisiran [14]	Small interfering RNA	ORION	NCT02597127	3	Binding to PCSK9 mRNA and silencing the expression of the target PCSK9 gene
**ASO**	AZD8233 [15]	Antisense oligonucleotide	ETESIAN	NCT04641299	2b	Inhibiting the gene transcript of a target protein PCSK9 in the nucleus
**Vaccines**	AT04A [16] AT06A [16]	Vaccine	-	NCT02508896	1	Leveraging the immune system to counteract PCSK9
			-	NCT02508896	1	
	VXX-401 [17]		-	NCT05762276	1	
**Oral PCSK9 inhibitors**	AZD0780	Antisense oligonucleotide	PURSUIT	NCT06173570 **	2b	Suppressing the expression of the PCSK9 gene
	MK-0616	Macrocyclic peptide	-	NCT05952856 ** NCT05952869 ** NCT06008756 **	3	Binding to PCSK9 and inhibiting the combination between PCSK9 and LDLR
**Gene therapy**	VERVE-101 [18]	Gene editing technique	VT-1001	NCT05398029 **	1	Disrupting target PCSK9 gene via CRISPR

Abbreviations: PCSK9, proprotein convertase subtilisin/kexin 9; CRISPR, clustered regularly interspaced short palindromic repeats. * development terminated; ** ongoing trials.
